# Supplementary material for: A Quorum Sensing-Regulated Type VI Secretion System Containing Multiple Nonredundant VgrG Proteins Is Required for Interbacterial Competition in Chromobacterium violaceum
Source: Microbiol Spectr. 2022 Jul 25;10(4):e01576-22. doi: 10.1128/spectrum.01576-22 (PMC9430734; doi:10.1128/spectrum.01576-22)
Supplement: Supplemental file 1 — Supplemental material. Download spectrum.01576-22-s0001.pdf, PDF file, 1.0 MB [file spectrum.01576-22-s0001.pdf]

SUPPLEMENTAL MATERIAL

Table S1. Bacterial strains and plasmids

Bacterial strain or plasmid	Description	Reference
Escherichia coli		
DH5α	Strain for cloning purposes	(1)
S17-1	Strain for plasmid mobilization	(2)
BL21(DE3)	Strain for protein expression	Novagen
Chromobacterium violaceum		
ATCC 12472	Wild type (Sequenced genome)	(3)
Δ*clpV* (CV_3965)	WT strain with CV_3965 gene deleted	This work
Δ*hcp* (CV_3977)	WT strain with CV_3977 gene deleted	This work
Δ*vipA* (CV_3979)	WT strain with CV_3979 gene deleted	This work
Δ*vgrG*1 (CV_3986)	WT strain with CV_3986 gene deleted	This work
Δ*vgrG*2 (CV_3975)	WT strain with CV_3975 gene deleted	This work
Δ*vgrG*3 (CV_1432)	WT strain with CV_1432 gene deleted	This work
Δ*vgrG*4 (CV_1233)	WT strain with CV_1233 gene deleted	This work
Δ*vgrG*5 (CV_0023)	WT strain with CV_0023 gene deleted	This work
Δ*vgrG*6 (CV_0016)	WT strain with CV_0016 gene deleted	This work
Δ*vgrG*1-2	WT strain with CV_3986 and CV_3975 genes deleted	This work
Δ*vgrG*1-3	WT strain with CV_3986, CV_3975 and CV_1432 genes deleted	This work
Δ*vgrG*1-4	WT strain with CV_3986, CV_3975, CV_1432 and CV_1233 genes deleted	This work
Δ*vgrG*1-5	WT strain with CV_3986, CV_3975, CV_1432, CV_1233 and CV_0023 genes deleted	This work
Δ*vgrG*1-6	WT strain with CV_3986, CV_3975, CV_1432, CV_1233, CV_0023 and CV_0016 genes deleted	This work
Δ*cviR*	WT strain with CV_4090 gene deleted	(4)
Δ*cviI*	WT strain with CV_4091 gene deleted	(5)
WT(pSEVA)	WT strain containing the empty vector pSEVA	This work
Δ*vgrG*3(pSEVA)	Δ*vgrG*3 strain containing the empty vector pSEVA	This work
Δ*vgrG*3(*vgrG*3)	In trans complementation of *vgrG*3 with pSEVA vector in Δ*vgrG*3	This work
WT(pMR20)	WT strain containing the empty vector pMR20	This work
Δ*hcp*(*hcp*)	In trans complementation of *hcp* with pMR20 vector in Δ*hcp*	This work
Δ*vipA*(*vipA*)	In trans complementation of *vipA* with pMR20 vector in Δ*vipA*	This work

Δ*vgrG*1-6(*vgrG*1)	In trans complementation of *vgrG*1 with pMR20 vector in the sextuple mutant (Δ*vgrG*1-6)	This work
Δ*vgrG*1-6(*vgrG*2)	In trans complementation of *vgrG*2 with pMR20 vector in the sextuple mutant (Δ*vgrG*1-6)	This work
Δ*vgrG*1-6(*vgrG*3)	In trans complementation of *vgrG*3 with pMR20 vector in the sextuple mutant (Δ*vgrG*1-6)	This work
Δ*vgrG*1-6(*vgrG*4)	In trans complementation of *vgrG*4 with pMR20 vector in the sextuple mutant (Δ*vgrG*1-6)	This work
Δ*vgrG*1-6(*vgrG*5)	In trans complementation of *vgrG*5 with pMR20 vector in the sextuple mutant (Δ*vgrG*1-6)	This work
Δ*vgrG*1-6(*vgrG*6)	In trans complementation of *vgrG*6 with pMR20 vector in the sextuple mutant (Δ*vgrG*1-6)	This work
WT(pCV_3981)	WT strain containing the promoter of CV_3981 with *lacZ* fusion	This work
Δ*cviR*(pCV_3981)	Δ*cviR* strain containing the promoter of CV_3981 with *lacZ* fusion	This work
WT(pCV_3982)	WT strain containing the promoter of CV_3982 with *lacZ* fusion	This work
Δ*cviR*(pCV_3982)	Δ*cviR* strain containing the promoter of CV_3982 with *lacZ* fusion	This work
WT(p*vgrG*1)	WT strain containing the promoter of *vgrG*1 with *lacZ* fusion	This work
WT(p*vgrG*2)	WT strain containing the promoter of *vgrG*2 with *lacZ* fusion	This work
WT(p*vgrG*3)	WT strain containing the promoter of *vgrG*3 with *lacZ* fusion	This work
WT(p*vgrG*4)	WT strain containing the promoter of *vgrG*4 with *lacZ* fusion	This work
WT(p*vgrG*5)	WT strain containing the promoter of *vgrG*5 with *lacZ* fusion	This work
WT(p*vgrG*6)	WT strain containing the promoter of *vgrG*6 with *lacZ* fusion	This work
Δ*cviR*(p*vgrG*3)	Δ*cviR* strain containing the promoter of *vgrG*3 with *lacZ* fusion	This work
Δ*cviR*(p*vgrG*4)	Δ*cviR* strain containing the promoter of *vgrG*4 with *lacZ* fusion	This work
WT(*vipA_sfGFP*)	WT with *vipA_sfGFP* cloned into pJN105 vector for L-arabinose induction	This work
Δ*clpV*(*vipA_sfGFP*)	Δ*clpV* with *vipA_sfGFP* cloned into pJN105 vector for L-arabinose induction	This work
Δ*hcp*(*vipA_sfGFP*)	Δ*hcp* with *vipA_sfGFP* cloned into pJN105 vector for L-arabinose induction	This work
Δ*vgrG*3(*vipA_sfGFP*)	Δ*vgrG*3 with *vipA_sfGFP* cloned into pJN105 vector for L-arabinose induction	This work
Δ*vgrG*1-3(*vipA_sfGFP*)	Δ*vgrG*1-3 with *vipA_sfGFP* cloned into pJN105 vector for L-arabinose induction	This work

Δ*vgrG*1-6(*vipA_sfGFP*)	Δ*vgrG*1-6 with *vipA_sfGFP* cloned into pJN105 vector for L-arabinose induction	This work
Δ*cviR*(*vipA_sfGFP*)	Δ*cviR* with *vipA_sfGFP* under L-arabinose induction	This work
Δ*cviI*(*vipA_sfGFP*)	Δ*cviI* with *vipA_sfGFP* under L-arabinose induction	This work
Δ*vgrG*3(*vgrG*3-HA)	Δ*vgrG*3 in trans complemented with pMR20 expressing *vgrG*3 gene fused to hemagglutinin tag at C-terminal portion	This work
**Plasmids**		
pNPTS138	Suicide vector containing oriT, Km^r, *sacB*	D. Alley
pJN105	Broad-host-range vector, araC-PBAD cassete; Gm^r	(6)
pMR20	Broad-host-range low-copy vector containing oriT, Tet^R	(7)
pET-15b	His-tagged protein expression vector; Amp^r	Novagen
pGEM-T easy	Cloning plasmid; Amp^R	Promega
pRK*lacZ*290	Vector containing promoterless *E. coli lacZ.* Tet^R	(8)
pSEVA221	Broad-host-range vector, Km^R, *oriRK2, oriT.*	(9)

1. Hanahan D. 1983. Studies on transformation of *Escherichia coli* with plasmids. J Mol Biol 166(4):557-580.
2. Simon R, Priefer U, Puhler A. 1983. A broad host range mobilization system for *in vivo* genetic engineering: transposon mutagenesis in Gram-negative bacteria. Bio/Technology 1:784–791.
3. Brazilian National Genome Project Consortium. 2003. The complete genome sequence of *Chromobacterium violaceum* reveals remarkable and exploitable bacterial adaptability. Proc Natl Acad Sci U S A 100(20)**:**11660-11665.
4. Batista JH, Leal FC, Fukuda TTH, Alcoforado Diniz J, Almeida F, Pupo MT, da Silva Neto JF. 2020. Interplay between two quorum sensing-regulated pathways, violacein biosynthesis and VacJ/Yrb, dictates outer membrane vesicle biogenesis in *Chromobacterium violaceum*. Environ Microbiol 22(6):2432–2442.
5. Barroso KCM, Previato-Mello M, Batista BB, Batista JH, da Silva Neto JF. 2018. EmrR-Dependent Upregulation of the Efflux Pump EmrCAB Contributes to Antibiotic Resistance in *Chromobacterium violaceum*. Front Microbiol 9:2756.
6. Newman JR, Fuqua C. 1999. Broad-host-range expression vectors that carry the L-arabinose-inducible *Escherichia coli araBAD* promoter and the *araC* regulator. Gene 227(2):197-203.
7. Roberts RC, Toochinda C, Avedissian M, Baldini RL, Gomes SL, Shapiro L. 1996. Identification of a *Caulobacter crescentus* operon encoding *hrcA*, involved in negatively regulating heat-inducible transcription, and the chaperone gene *grpE*. J Bacteriol 178(7):1829-1841.
8. Gober JW, Shapiro L. 1992. A developmentally regulated *Caulobacter* flagellar promoter is activated by 3' enhancer and IHF binding elements. Mol Biol Cell 3(8):913-26.
9. Silva-Rocha R, Martínez-García E, Calles B, Chavarría M, Arce-Rodríguez A,de Las Heras A, Páez-Espino AD, Durante-Rodríguez G, Kim J, Nikel PI,Platero R, de Lorenzo V. 2013. The Standard European Vector Architec-ture (SEVA): a coherent platform for the analysis and deployment of complex prokaryotic phenotypes. Nucleic Acids Res 41:D666–D675.

Table S2. Primers used in this work

**Name**	**Sequence 5' - 3' ***	**Description**
**Construction of mutant strains**		
CV_3965 del1<br>CV_3965 del2	GATCATAAGCTTGATTGATGGGGCGTGGGAAG<br>GATCATGAATTCCGTCAACCGGTCTATCAGGG	767 pb, *HindIII/EcoRI*. CV_3965 deletion
CV_3965 del3<br>CV_3965 del4	GATCATGAATTCCGTATGGCCAGCGGCAAACC<br>GATCATGTCGACGCTTCAGTTCCTTGACCCGG	652 pb, *EcoRI/SalI*. CV_3965 deletion
CV_3977 del1<br>CV_3977 del2	GATCATAAGCTTGAAGTGGCGCTGAAGTGCCC<br>GATCATCTGCAGGCTTTCGCCAGGAATGCCATC	633 pb, *HindIII/PstI*. CV_3977 deletion
CV_3977 del3<br>CV_3977 del4	GATCATCTGCAGGGCAATGTCAGCGCCGGTTG<br>GATCATGGATCCGAGAGCTAAGGTCCATGGCG	687 pb, *PstI/BamHI*. CV_3977 deletion
CV_3979 del1<br>CV_3979 del2	GACTATGGGCCCATTCCCTGTCGCGCAGCCTG<br>GACTATGGATCCGCCGATCTCGACGTCATAGG	607 pb, *ApaI/BamHI*. CV_3979 deletion
CV_3979 del3<br>CV_3979 del4	GACTATGGATCCGACGAAGTGGTGCGCGATAC<br>GACTATGAATTCCCCACCAGCGCGGCGTAAG	547 pb, *BamHI/EcoRI*. CV_3979 deletion
CV_3986 del1<br>CV_3986 del2	TACCGGAAGCTTTGTACTTCGCGCTATTGGCC<br>TACCGGGGATCCGCTGGTCAGATCCATGATCG	606 pb, *HindIII/BamHI*. CV_3986 deletion
CV_3986 del3<br>CV_3986 del4	TACCGGGGATCCCTGAATGCGATCAAATCCGGC<br>TACCGGGAATTCTTGGCCAATGCATCGGGCTC	663 pb, *BamHi/EcoRI*. CV_3986 deletion
CV_3975 del1<br>CV_3975 del2	TACCGGAAGCTTGCCCCGGGCGGGTTTTTAAG<br>TACCGGGGATCCGTCCATGGCGCAATCTATCCG	598 pb, *HindIII/BamHI*. CV_3975 deletion
CV_3975 del3<br>CV_3975 del4	TACCGGGGATCCGTGGCCGCCAAATGAAGCCG<br>TACCGGGAATTCCCAACAGGTGGTTTTGCCGC	660 pb, *BamHI/EcoRI*. CV_3975 deletion
CV_1432 del1<br>CV_1432 del2	TACCGGAAGCTTCGGCCGCCAAACTACTAGTC<br>TACCGGGGATCCGTCAAGGTCCATCGATTGTCC	626 pb, *HindIII/BamHI*. CV_1432 deletion
CV_1432 del3<br>CV_1432 del4	TACCGGGGATCCTTCCCCTTCGTAGAGCAGCC<br>TACCGGGAATTCCGGCAGGAACGACGCCAATC	603 pb, *BamHI/EcoRI*. CV_1432 deletion
CV_1233 del1<br>CV_1233 del2	TACCGGAAGCTTCCAGGCTTCCAGATACTGCG<br>TACCGGGGATCCACTGGCTAGCAATGCGTTCAG	611 pb, *HindIII/BamHI*. CV_1233 deletion
CV_1233 del3<br>CV_1233 del4	TACCGGGGATCCCATTTCAACATGGTGCCGTCG<br>TACCGGGAATTCCCCTCCAGCGACTTCATTTC	583 pb, *BamHI/EcoRI*. CV_1233 deletion
CV_0023 del1<br>CV_0023 del2	TACCGGAAGCTTTGGCATGTTGGCAACAGGCG<br>TACCGGGGATCCCAGATCCATCGGCGTTCCAG	593 pb, *HindIII/BamHI*. CV_0023 deletion
CV_0023 del3<br>CV_0023 del4	TACCGGGGATCCCCGACGCTCCCCAAAGACAA<br>TACCGGGAATTCTGCATAGAACGGTGTGCCGG	657 pb, *BamHI/EcoRI*. CV_0023 deletion
CV_0016 del1<br>CV_0016 del2	TACCGGGGGCCCGAGATGCTAAGGCCGGTGAC<br>TACCGGGGATCCGAAGCTGGAGAGGAGGCTGT	592 pb, *ApaI/BamHI*. CV_0016 deletion
CV_0016 del3<br>CV_0016 del4	TACCGGGGATCCGCGAGTACCAACTTGCCCTTC<br>TACCGGGTCGACCTCCCCATGAAATGGATGGCG	632 pb, *BamHI/SalI*. CV_0016 deletion
**Construction of complemented strains**		
CV_3977 Comp Fw<br>CV_3977 Comp Rv	GATCATCTGCAGGAAGCCAAGGCCAAGTACCC<br>GATCATGAGCTCCGCAAGACAAGGCGACAACC	852 pb, *PstI/SacI*. Product with *hcp* gene and its promoter region
CV_3979 Comp Fw<br>CV_3979 Comp Rv	GACTATGAATTCGGGGCGGCAATTGAACGATC<br>GACTATGAGCTCCGATGATGCTGTCGAGCAGG	699 pb, *EcoRI/SacI*. Product with *vipA* gene and its promoter region
CV_3979 Over Fw<br>CV_3979_sfgfp Rv	GACTATGAATTCGAACATCTTGTAGAGAAGAGCC<br>GACTATCTGCAGGGGGGGGGGCGGCGGCGCTGCT CGCCTCCTTCTTTCT	526 pb, *PstI/EcoRI*. *vipA* full gene plus 3 Gly x 3 Ala linker
sfGFP_Fw<br>sfGFP_Rv	GACTATCTGCAGATGTCGAAAGGAGAAGAACTGT<br>GACTATGAGCTCTTTATACAGTTCATCCATTCCATG	714 pb, *PstI/SacI*. Product with super folder GFP for cloning with *vipA* into pJN105
CV_3977 Exp Fw<br>CV_3977 Exp Rv	GATCACATATGGCTTTTGATGCATTTCTGAAAATC<br>GATCAGGATCCTTAGGCGATTACTTTGTTCGCG	498 pb, *NdeII/BamHI*. Product with *hcp* gene for heterologous expression
CV_3986 Comp Fw<br>CV_3986 Comp Rv	TACCGGGGTACCCTCGATCGAGCTGCATGAGC<br>TACCGGGGATCCCATCCCCCTTGTCCATTGCG	2861 pb, *KpnI/BamHI*. CV_3986 complementation
CV_3975 Comp Fw<br>CV_3975 Comp Rv	TACCGGGGTACCCCGCCAATGTGCGCGATTAC<br>TACCGGGGATCCGGTGTCTTGCAACAGCATGC	3019 pb, *KpnI/BamHI*. CV_3975 complementation
CV_1432 Comp Fw<br>CV_1432 Comp Rv	TACCGGGGTACCCGGGTCTCTCATGATCTTGCC<br>TACCGGGGATCCGCTATGGGTGCGGTCATGGC	3554 pb, *KpnI/BamHI*. CV_1432 complementation

CV_1432-HA_TagCompRv	TACCGGGGATCCTCAAGCGTAGTCTGGGACGTCGTATGGGTAAGCGTAGTCTGGGACGTCGTATGGGTAGAACAGTTTGGGCAGGCTGGG	Primer used with CV_1432 Comp Fw. 3568 pb, *BamHI.* CV_1432 fused to HA epitope complementation
CV_1233 Comp Fw<br>CV_1233 Comp Rv	TACCGGGGTACCCCAGGCTTCCAGATACTGCG<br>TACCGGGAATTCGACGCGGTTTATGCTTGGCC	3506 pb, *KpnI/EcoRI.* CV_1233 complementation
CV_0023 Comp Fw<br>CV_0023 Comp Rv	TACCGGGGTACCCGGTCCCAAGTCTTGCTGTG<br>TACCGGGGATCCCTCGGGAAGAGAACGGCATC	2952 pb, *KpnI/BamHI.* CV_0023 complementation
CV_0016 Comp Fw<br>CV_0016 Comp Rv	TACCGGGGTACCGAGATGCTAAGGCCGGTGAC<br>TACCGGGGATCCCCGGCTCTCTACCAGTAATC	3234 pb, *KpnI/BamHI.* CV_0016 complementation
**B-galactosidase assay**		
pCV_3981Fw<br>pCV_3981Rv	GATCATGGATCCGCCAAGCACATTCTCGATTGG<br>GATCATAAGCTTGCGATTGTCGCCTGTTCCAC	531 pb, *BamHI/HindIII.* Promoter region of CV_3981 for pRK*lacZ*290 cloning
pCV_3982Fw<br>pCV_3982Rv	GATCATAAGCTTGCCAAGCACATTCTCGATTGG<br>GATCATGGATCCGCGATTGTCGCCTGTTCCAC	531 pb, *HindIII/BamHI.* Promoter region of CV_3982 for pRK*lacZ*290 cloning

***** Underlined letters indicate the restriction enzyme recognition sites

Table S3. Bacteria strains used as prey in interbacterial competition assay

**Bacteria**	**Strain**	**Abbreviation ***
Burkholderia cepacia	ATCC 17759	Bc
Escherichia coli	ATCC 25922	Ec
Pseudomonas aeruginosa	ATCC 27853	Pa
Staphylococcus aureus	ATCC 29313	Sa
Salmonella typhimurium	ATCC 14028	St
Stenotrophomonas maltophilia	ATCC 13637	Sma
Enterobacter cloacae	ATCC 13047	Ecl
Shigella flexneri	ATCC 12022	Sf
Enterococcus faecium	NCTC 13047	Ef
Klebsiella pneumoniae	ATCC 13883	Kp
Klebsiella pneumoniae	ATCC BAA-1705	Kpn
Enterococcus faecalis	ATCC 4083	Efa
Shigella sonnei	ATCC 25931	Ss
Pseudomonas aeruginosa	PAO1	Pao

*Abbreviation referring to **Figure 2A**

Table S4. Proteins identified in coimmunoprecipitation assay of WT(pMR20) and Δ*vgrG*3(*vgrG*3-HA)

**WT(pMR20)**						
**Accession***	**Description**	**mW (Da)**	**Peptides**	**Coverage (%)**	**Products**	**Amount (fmol)**
**Q7M7F1**	Elongation factor Tu	43045	92	86,3636	974	32,4532
Q7NQE7	DNA-directed RNA polymerase subunit beta'	155194	86	63,8252	640	2,0263
**Q7NQF0**	Elongation factor G	76958	60	79,7994	442	1,4946
**Q7NQG4**	50S ribosomal protein L5	20292	14	46,3687	70	0,3518
**Q7NQG5**	30S ribosomal protein S14	11589	7	38,6139	18	0,4214
**Q7NQG8**	50S ribosomal protein L18	12765	9	64,1026	52	0,5157
**Q7NQG9**	30S ribosomal protein S5	18207	14	47,6744	81	0,1538
Q7NQM5	Aspartate ammonia-lyase	50287	24	76,8737	218	0,9885
**Q7NQX1**	60 kDa chaperonin 2	57382	40	69,7802	347	1,5874
Q7NR97	Nudix hydrolase domain-containing protein	23223	10	66,8269	105	0,1679
**Q7NUY8**	Trigger factor	48546	34	81,3793	262	1,2455
Q7NV09	Uncharacterized protein	6615	3	68,3333	38	0,0807
Q7NV22	Probable transmembrane protein	13147	4	71,7742	53	0,0143
Q7NVZ4	30S ribosomal protein S2	27112	18	69,1358	111	1,3853
Q7NWY4	Phosphonate metabolism protein PhnH	20435	6	54,2105	66	0,0398
Q7NX50	Probable transcriptional regulator_ MerR family	15218	9	58,4615	100	0,0483
Q7NZ92	Uncharacterized protein	10198	4	43,8202	40	0,5778
**Q7P095**	ATP synthase subunit beta	50024	40	81,0753	434	2,0939
**Q7P097**	ATP synthase subunit alpha	54676	36	69,0661	327	2,4705
**Q7P0N9**	Acetyltransferase component	56484	19	49,0975	210	1,8117
Q7P0P0	Pyruvate dehydrogenase E1 component	99230	67	81,2852	584	2,3749
**Δ*vgrG*3(*vgrG*3-HA)**						
**Accession**	**Description**	**mW (Da)**	**Peptides**	**Coverage (%)**	**Products**	**Amount (fmol)**
**Q7M7F1**	Elongation factor Tu	43045	54	72,2222	714	45,6856
**Q7NQF0**	Elongation factor G	76958	39	55,5874	313	2,9557
Q7NQF9	50S ribosomal protein L16	15401	4	30,4348	49	5,6009
Q7NQG2	50S ribosomal protein L14	13409	15	51,6393	89	3,7829
**Q7NQG4**	50S ribosomal protein L5	20292	16	65,3631	108	0,739
**Q7NQG5**	30S ribosomal protein S14	11589	5	45,5446	56	4,4619
**Q7NQG8**	50S ribosomal protein L18	12765	7	52,9915	88	9,9647
**Q7NQG9**	30S ribosomal protein S5	18207	18	59,8837	149	3,5319
Q7NQH0	50S ribosomal protein L30	6780	5	37,7049	33	1,1832
**Q7NQX1**	60 kDa chaperonin	57382	18	46,1538	164	2,9832
Q7NQZ9	VgrG1 (CV_3986)	93995	27	41,7431	314	0,2691
Q7NR08	Hcp (CV_3977)	17881	8	53,3333	81	0,9134
Q7NR10	VgrG2 (CV_3975)	94022	20	35,8945	255	1,3435
Q7NRL5	30S ribosomal protein S21	8490	2	28,5714	19	1,6007

Q7NRT4	30S ribosomal protein S9	14356	4	36,1538	44	2,1704
Q7NRV5	30S ribosomal protein S16	9536	7	59,0361	84	8,6455
Q7NU57	Uncharacterized protein (CV_2846)	7517	2	45,4545	58	77,9891
**Q7NUY8**	Trigger factor	48546	15	42,7586	187	2,7161
Q7NY43	VgrG3 (CV_1432)	107856	29	42,1782	363	2,6193
**Q7P095**	ATP synthase subunit beta	50024	27	51,1828	324	3,949
**Q7P097**	ATP synthase subunit alpha	54676	18	33,8521	217	4,4851
**Q7P0N9**	Acetyltransferase component	56484	12	32,3105	147	1,1308
Q7P238	VgrG5 (CV_0023)	94125	17	29,9771	247	1,2663

*Accessions highlighted in bold indicate proteins identified in both assays and are probably contaminant proteins with affinity to the magnetic beads.

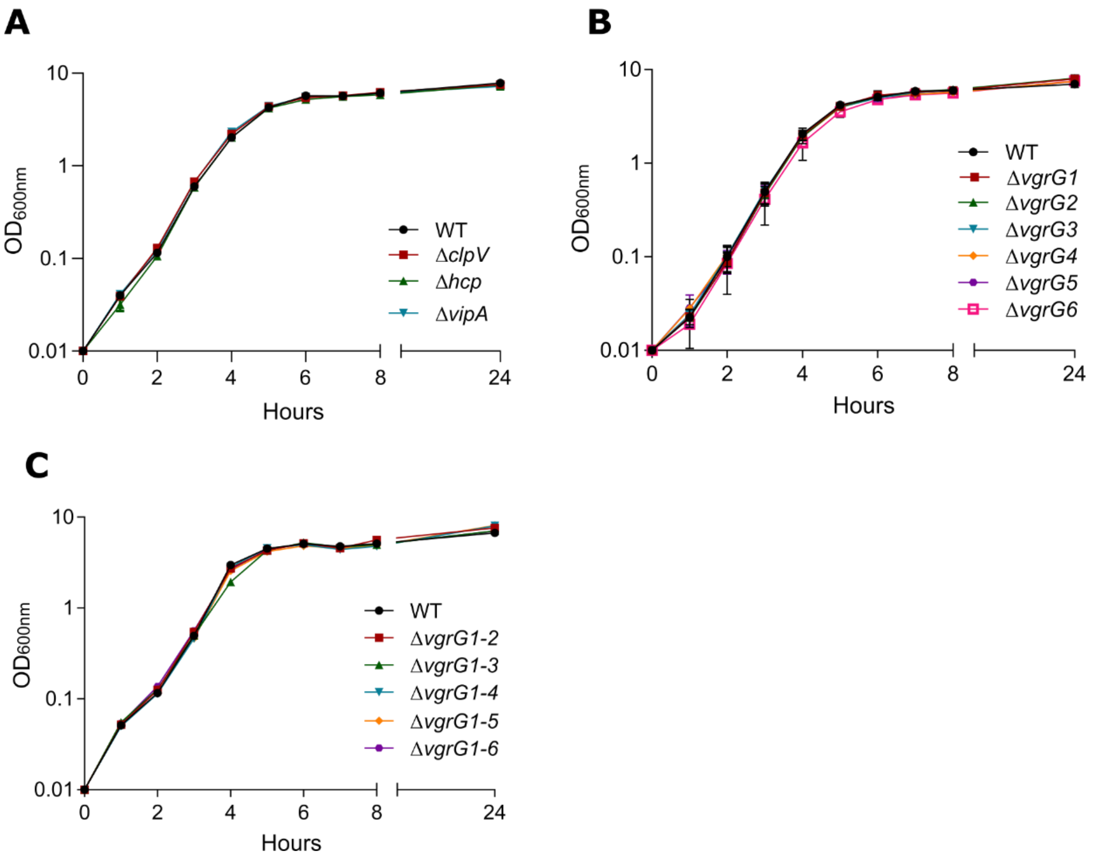

Fig S1 Growth curves of the indicated strains grown in LB medium. (A) Mutants for T6SS core components. (B) Single mutant strains for each *vgrG*. (C) Sequential *vgrG* mutants. None of these strains showed any growth delay compared to the WT strain.

A

	VgrG1 (CV_3986)	VgrG2 (CV_3975)	VgrG3 (CV_1432)	VgrG4 (CV_1233)	VgrG5 (CV_0023)	VgrG6 (CV_0016)
**VgrG1 (CV_3986)**	100					
**VgrG2 (CV_3975)**	93.43	100				
**VgrG3 (CV_1432)**	93.46	92.20	100			
**VgrG4 (CV_1233)**	76.46	76.23	71.06	100		
**VgrG5 (CV_0023)**	84.36	84.27	83.55	82.50	100	
**VgrG6 (CV_0016)**	81.68	83.20	81.80	80.18	93	100

B

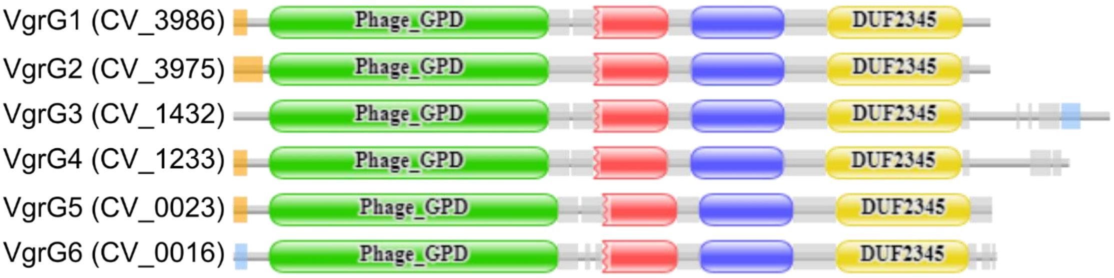

C

```
VgrG1           MDLTSLLSSFASAFTQDQRLLTLELGSGQVAAEQLLPQSLNGEEGVSQAYRYQLTCLSPD     60
VgrG2           MDLSSLLASFASAFNQDQRLLTLELGSGQVAAEQLLPQSLNGEEGVSQAYRYQLTCLSPD     60
VgrG3           MDLDTLLASFASAFNQDQRLLTLELGSGQVAAEQLLPQSLNGEEGVSQAYRYQLTCLSPD     60
VgrG4           MDLNALLASFASAFSQEQRLISLEFGGGHIASEQLLPLSLDGEEGISRSYRYTLTCLSPD     60
VgrG5           MDLNALLSSFASAFGQDHRPLTLELGSGQIAAEQLLPLSLDGEEGLSRAYRYTVTCLSPD     60
VgrG6           MDLNSLLSSFASAFNQEQRLLTLELGTGQIAAEQLLPLSLDGEEGVSRAYRYTVTCLSPD     60
                *** :**:****** *::* ::**:* *::*:***** **:****:*::*** :******

VgrG1           GAIELKTLLGQAARIGIADAQGQETIRCGVVSQARLMGSDGGFAQYGLTIEPPIALLRHR    120
VgrG2           GAIELKTLLGQAARIGIADAQGQETIRCGVVSQARLMGSDGGFAQYGLTIEPPIALLRHR    120
VgrG3           GAIELKTLLGQAARIGIADAQGQETIRCGVVSQARLMGSDGGFAQYGLTIEPPIALLRHR    120
VgrG4           GNIELKTLLGQPARIGIQDADGDETIRCGVVSQAKLAGSDGGFAKYELTIEPPFALLRHR    120
VgrG5           GHIELKTLLGQPARIGIQDADGDETIRCGVVSQARLAGADGGFARYELTIEPPFALLRHR    120
VgrG6           GHIELKTLLGQPARIGIQDADGDETIRCGMVSQARLAGADGGFARYELTIEPPFALLRHR    120
                * ********* ***** **:*:******:****:* *:*****:* ******:******

VgrG1           KTSRVFQDLSVPDIVQQIVHEHQAANPVFARAQSIEFKVGPAQPRSYCLQYREDDFSFIV    180
VgrG2           RTSRVFQDLSVPDIVQQIVHEHQAANPVFARAQTVEFKVGPAQPRSYCLQYREDDFSFIV    180
VgrG3           KTSRVFQDLSVPDIVQQIVHEHQAANPVFARAQSIEFKVGPAQPRSYCLQYREDDFSFIV    180
VgrG4           RTSRVFQDLSVPDIVKQIIGEHQAANPAFARVQTLGIQVKPAQPRSYTLQYRESDFDFIV    180
VgrG5           KTSRVFQDLSVPDIVKQILAEHQAANPAFARTQTLDIQVKPAPPRSYTLQYRESDFDFIV    180
VgrG6           KTSRVFQDLSVPDIVKQILAEHQAANPAFARAQTLDIQVRPAPPRSYTLQYRESDFDFIV    180
                :**************:**: *******.***.*:: ::* ** **** *****.**.***

VgrG1           RLLHEEGYAWRFEHVD-----------GDSPQVKLVVFDDAYSLPPAEVERVRFHRSDAT    229
VgrG2           RLLHEEGYAWRFEHVD-----------GDAPQVKLAVFDDAYSLPPAEVERVRFHRSDAT    229
VgrG3           RLLHEEGYAWRFEHVD-----------GDSPQVKLVVFDDAYSLPPAEVERVRFHRSDAT    229
VgrG4           RLIHEEGYAWRFLHLD-----------DDGPQVELAIFDDAHSLPGSKLERVRFHRSDAS    229
VgrG5           RLLHEEGYAWRFVHLDEVTERGSEASRGNSPQVELAVFDDALSLPAAKLERVRFHRSDAT    240
VgrG6           RLLHEEGYAWRFVHLDEVTERGSEASRGNSPQVELAVFDDAHSLPAAKLERVRFHRSDAT    240
                **:********* *:*           .:.***:*.:**** *** :::**********:

VgrG1           EEEDGLTDWSAARQIVPGNVALATFDYQPVSTQHTGDSSQIDQGPGGQALQSSLQDYDPQ    289
```

```
VgrG2           EEEDGLTDWSAARQIVPGNVALATFDYQPVSTQHTGDSSQIDQGPGGQALQSSLQDYDPQ    289
VgrG3           EEEDGLTDWSAARQIVPGNVALATFDYQPVSTQHTGDSSQIDQGPGGQALQSSLQDYDPQ    289
VgrG4           EEEDGLTGWEAARQIVPGGTALASYDYKSVYALQSGDQSRIDQGQAGQSLQSSLRHYDAP    289
VgrG5           EEEDGLTEWQAARQIVPGGTALASYDYKPVYSQQNRDDSHIDQGQTGQALQSSLTQYDAP    300
VgrG6           EEEDGLTEWQAAHQIVPGGTTLVSYDYKPVYSQQNRDDSHIDQGQTGQALQSSLTQYDAP    300
                ******* *.**:*****..:*.::**: * : :. *.*:****  **:***** .**

VgrG1           GLYYAGDAEQLSHYARLRQQAHDLQAKTFEGAGSIRGLTAGQWFRLDDHPAHEADSHEQR    349
VgrG2           GLYYAGDAEQLSHYARLRQQAHDLQAKSFEGAGSIRGLTAGQWFRLDDHPAHEADSHEQR    349
VgrG3           GLYYAGDAEQLSHYARLRQQAHDLQAKTFEGAGSIRGLTAGQWFRLDDHPAHEADSHEQR    349
VgrG4           GLYYAGDEEQLGQYAALRQQANDLQAKSFSGTGSVRGLLPGEWFRLDEHPAHDSDAPASR    349
VgrG5           GLYYADDEEQLGRYAQLRQQANDLQAKSFSGSGSVRGLLPGDWFRLDDHPAHDGDAAENR    360
VgrG6           GLYYAGDEEQLGRYAQLRQQANDLQAKSFSGSGSVRGLLPGDWFRLDDHPAHDGDAAENR    360
                *****.* ***.:** *****:*****:*.*:**:***  *:*****:****:.*:  .*

VgrG1           EFVVTGQTLQVRNNLPTDLQSILPTGDKADAPFRTRIQAQRRGIPLTPAYAGTEHAKPKS    409
VgrG2           EFVVTGQTLQVRNNLPTDLQSILPTDDQTDAPFTTRIQAQRHGIPLTPAYAGTEHAKPKS    409
VgrG3           EFVVTGQTLQVRNNLPTDLQSILPTGDKADAPFRTRIQAQRRGIPLTPAYAGTEHAKPKS    409
VgrG4           EFVVTGQSFQARNNLPTDLAQHIGAE-QDAAPFTTSIQAQRRGIPLTPAYAGTAHAKPTS    408
VgrG5           EFVVTGQSFQVRNNLPQDLAQHIGTE-SDATPFATSIQAQRRGIPLTPAYAGTEHAKPKS    419
VgrG6           EFVVTGQSFQVRNNLPQDLAQHIGTE-SDATPFATSIQAQRRGIPLTPAYAGTELAKPTS    419
                *******::*.***** ** . : :  .  :** * *****:***********  ***.*

VgrG1           RGVQTATVVGPAGEEVHTDQHGRIKVQFHWQRPDEHPTIGAALDDKSSCWLRVAMPSAGA    469
VgrG2           RGVQTATVVGPAGEEVHTDQHGRIKVQFHWQRPDEHPTIGAALDDKSSCWLRVAMPSAGA    469
VgrG3           RGVQTATVVGPAGEEVHTDQHGRIKVQFHWQRPDEHPTIGAALDDKSSCWLRVAMPSAGA    469
VgrG4           QGAQTATVVGPAGEEVHTDEQGRVKVQFHWQRQEEHPTIGAGMDDKSSCWLRVAMPSAGA    468
VgrG5           RGVQTATVVGPAGEEVHTDQHGRIKVQFHWQRPDEHPTIGAALDDKSSCWLRVAMPSAGA    479
VgrG6           LGVQTATVVGPAGEEVHTDQHGRIKVQFHWQRPDEHPTIGASLDDKSSCWLRVAMPSAGA    479
                 *.****************::**:******** :*******.:*****************

VgrG1           GWGHQFIPRIGQEVLVDFIEGDIDRPVITGVLYNGSHPTPDFSGAGSLPANKTLSGIKSK    529
VgrG2           GWGHQFIPRIGQEVLVDFIEGDIDRPVITGVLYNGSHPTPDFSGAGSLPANKTLSGIKSK    529
VgrG3           GWGHQFIPRIGQEVLVDFIEGDIDRPVITGVLYNGSHPTPDFSGAGSLPANKTLSGIKSK    529
VgrG4           AWGHQFIPRIGQEVLVNFIEGDIDRPLITGVLYNGSHPPPDFSGAGALPANKTLSGIKSK    528
VgrG5           GWGHQFIPRIGQEVLVDFIEGDIDRPVITGVLYNGSHPTPDFSGAGALPANKTLSGIKSK    539
VgrG6           GWGHQFIPRIGQEVLVDFIEGDIDRPVITGVLYNGSHPTPDFSGAGSLPANKTLSGIKSK    539
                .***************:*********:*********** *******:*************

VgrG1           EHQGGGYNELLFDDTPGEVRAKLSSEPGKTQLNQGYLAHPRSNGKAQPRGDGFELRTDHH    589
VgrG2           EHQGGGYNELLFDDTPGEVRAKLSSEPGKTQLNQGYLAHPRSNGKALPRGDGFELRTDHH    589
VgrG3           EHQGGGYNELLFDDTPGEVRAKLSSEPGKTQLNQGYLAHPRSNGKAQPRGDGFELRTDHH    589
VgrG4           EHQGGAYNELLFDDTPGEVRAKLSSELGKTQLNQGFLTHPRSNGKAQPRGEGFELRTDQH    588
VgrG5           EHQGGGYNELLFDDTPGEVRAKLSSEPGKTQLNQGYLAHPRSNGKAQPRGDGFELRTDHH    599
VgrG6           EHQGGGYNELLFDDTPGEVRAKLSSEPGKTQLNQGYLAHPRSNGKAQPRGDGFELRTDHH    599
                *****.******************** ********:*:******** ***:*******:*

VgrG1           GAIRAAHGLLLTTEAQNGASGKQLAREHAQSQLDAALSLSQALAETASGQLADTMETGPD    649
VgrG2           GAIRAAHGLLLTTEAQNGASGKQLAREHAQSQLDAALSLSQALAETASGQLADAMETGPD    649
VgrG3           GAIRAAHGLLLTTEAQNGASGKQLAREHAQSQLDAALSLSQALAETASGQLADTMETGPD    649
VgrG4           GAIRAAHGLLLSTEAQNGASGKQLAREHAQSQLQSAVALSQALAETATGQLADTMETGPD    648
VgrG5           GAIRAAHGLLLTTEAQNGASGKQLAREHAQSQLDAALSLSQALAETASGQLADAMETGPD    659
VgrG6           GAIRAAHGLLLTTEVQNGASGKQLAREHAQSQLDAALSLSQALAETASGQLADTMETGPE    659
                ***********:**.******************::*::*********:*****:*****:

VgrG1           EIGPDNAKAGKKTDGHLQHHADALKAWEAGSNTDKDGKTAKDQAGQQPLLVLSAPAGIAS    709
VgrG2           EIGPDNAKAGKKTDGHLQHHVDALKAWEAGSNTDKDGKTAKDQAGQQPLLVLSAPAGIAA    709
VgrG3           EIGPDNAKAGKKTDGHLQHHADALKAWEAGSNTDKDGKTAKDQAGQQPLLVLSAPAGIAS    709
VgrG4           GIGEDNAKAGKKAKGHMQHQLDALAAWEAGSNTDKDGKTAKGQAGQQPQLVLSGPAGIAS    708
VgrG5           EIGPDNAKAGKKTDGHLQHHADALKAWEAGSNTDKDGKTAKDQAGQQPLLVLSAPAGIAA    719
VgrG6           EIGPDNAKAGKKSDGHLQHHADALKAWEAGSNTDKDGKTAKDQAGQQPLLVLSAPAGIAA    719
                 ** ********:.**:**: *** ****************.****** ****.*****:

VgrG1           LTEQSQTVSAGQNLNLIAQRDANHTTGRRWLHNVGQHISLFVAGVKDKVALKLIAAKGKV    769
VgrG2           LTEQSQTVSAGQNLNLVAQRDANHTTGRRWIHNVGQHISLFVAGVKDKVALKLIAAKGKV    769
VgrG3           LTEQSQTVSAGQNLNLIAQRDANHTTGRRWLHNVGQHISLFVAGVKDKVALKLIAAKGKV    769
VgrG4           VTEQSQTLAAGANLNLVVQRDANHTTGRRWVHNVGKHISLFVAGIKDNTAMKLIAAKGKV    768
VgrG5           LTEQSHTVSAGQNLNLVAQRDANHTTGRRWLHNVGQHISLFVAGVKDKVALKLIAAKGKV    779
```

```
VgrG6      LTEQSHTVSAGQNLNLVAQRDANHTTGRRWIHNVGQHISLFVAGVKDKVALKLIAAKGKV    779
           :****:*::** ****:.************:****:********:**:.*:*********

VgrG1      QVQAQSDAMELTADKDVTIMSCKERIVVNAKDEILLTA-GGAYIRLKGGNIEVHAPGTVS    828
VgrG2      QVQAQSDAMELTADKDVTITSAKQTIHVNAKQEILLTS-GGAYIRLKDGKIELHAPGTIS    828
VgrG3      QVQAQSDAMELTADKELTITSAKGKVQIAASQEVLLTS-GGGYIRIAGGNIEIHCPSEVS    828
VgrG4      QILAQSDAMEWTADKDVTITSCKDSITIAAKEEILLNVGGGAYIRLAGGNIEVHCPGTVS    828
VgrG5      QVQAQSDAMELTADKDVTITSCKERIVVNAKQEILVTA-GGGYIRIAGGNIEVHCPGTVS    838
VgrG6      Q--AQSDAMELTADKDVAITSVKQKVQFNGKQEILLTS-GGAYVRIKDGKIELHAPGTVS    836
           *  ******* ****:::* * *  : . ..:*:*:.  **.*:*: .*:**:*.*. :*

VgrG1      VKAAQHKLSGPDNMTIPMPVFPGKQFCLQ---CMLNAIKSGSPLA-GQ------------    872
VgrG2      IKGASHNFSGPDQMNPPLPRFPNT-VCKQ---CMAQAFSQANPLV-AAK-----------    872
VgrG3      VKGASHSLSGPASLSTPTPAFAKPGTGNL---QVIHEFANGFSMN-QGKFVVTDALGVKH    884
VgrG4      VKGAQHNLSGPDKMTVAHPAFPKNIPKQNIGLKVNHSFVEGDHAVPGMPFKLYADGAMIK    888
VgrG5      VKGASHDLSGPASMNPPLPTLPKDKYTP----PNTHPFSE--------------------    874
VgrG6      FKGASHDWSGPASTNLPFPTLPQGDTPS----CQLAAIQHKSGMT-KK------------    879
           .*.*.*. *** . .   * :                :

VgrG1      ------------------------------------------------------------    872
VgrG2      ------------------------------------------------------------    872
VgrG3      KGVLDEAGKAMVHGLPVGPAQVDFLGRPHKDKADIFPFVEQ-PEAALQQMGETVKGKAMQ    943
VgrG4      QGVLDETGHLPVEHNVLTQKYTLELANGIKRHFNMVPSYAKPPHDELAN-GGINHGEQSD    947
VgrG5      ------------------------------------------------------------    874
VgrG6      ------------------------------------------------------------    879


VgrG1      ------------------------------------------------------------    872
VgrG2      ------------------------------------------------------------    872
VgrG3      ELGSTLGKTSPQLANAMGQASAMAGQAQQALALARNPQSAIPALAAKAKEVLASKAQGAL   1003
VgrG4      KLD--------QYQDFF------GGKGEQA------------------------------    963
VgrG5      ------------------------------------------------------------    874
VgrG6      ------------------------------------------------------------    879


VgrG1      -------      872
VgrG2      -------      872
VgrG3      PSLPKLF      1010
VgrG4      -------      963
VgrG5      -------      874
VgrG6      -------      879
```

Fig S2 The six VgrG proteins of *C. violaceum* show high sequence identity and similar domain organization. (A) Identity percentages shared among the six VgrG proteins of *C. violaceum*. The values were obtained with the Clustal Omega tool for multiple alignment. (B) Modular domains of VgrG proteins from analysis in the Pfam database. Green, red, and blue indicate the typical VgrG domains. All VgrGs have an additional C-terminal domain, DUF2345 (yellow). VgrG3 contains another additional region at C-terminus with low complexity (light blue). VgrG1, VgrG2, VgrG4, and VgrG5 have putative signal sequences at their N-termini (orange). (C) Multiple sequence alignment of VgrGs using the Clustal Omega tool.
